# Positive inotropic and chronotropic effects of amisulpride via stimulation of 5-HT_4_ serotonin receptors in the isolated atrium from mouse and humans

**DOI:** 10.1007/s00210-026-05219-7

**Published:** 2026-03-21

**Authors:** Joachim Neumann, Britt Hofmann, Ulrich Gergs

**Affiliations:** 1 Institute for Pharmacology and Toxicology, Medical Faculty, Martin-Luther-University Halle-Wittenberg, Magdeburger Straße 4, D-06097 Halle (Saale), Germany; 2 Department of Cardiac Surgery, Mid-German Heart Centre, University Hospital Halle, Ernst-Grube-Straße 40, D-06097 Halle (Saale), Germany

**Keywords:** Amisulpride, 5-HT_4_ serotonin receptor, Transgenic mice, Human atrium

## Abstract

Based on the chemical structure of amisulpride as a benzamide (4-amino-N-[(1-ethyl-2-pyrrolidinyl)-methyl]-5-(ethylsulfonyl)-2-methoxy-benzamide), we hypothesized that like many other benzamides, amisulpride might be an agonist or antagonist at the human cardiac 5-HT_4_ serotonin receptor. To test this hypothesis, we utilized isolated atria from transgenic adult mice with cardiac overexpression of the human 5-HT_4_ serotonin receptor (5-HT_4_-TG). In the presence of rolipram (100 nM, a phosphodiesterase IV inhibitor), amisulpride (10 µM) raised the force of contraction in isolated left atrial preparations and the beating rate in isolated spontaneously beating right atrial preparations from 5-HT_4_-TG but not from adult wild-type mice. The positive chronotropic effects of amisulpride in right atrial preparations from 5-HT_4_-TG were smaller than those of 1 µM serotonin and were reversed by 1 µM GR125487, an antagonist at the 5-HT_4_ serotonin receptor. Amisulpride (10 µM) raised the force of contraction in human right atrial preparations, obtained from patients undergoing cardiac surgery, only in the presence of 100 nM cilostamide, a phosphodiesterase III inhibitor. The positive inotropic effects of amisulpride in human right atrial preparations were reversed by 1 µM GR125487. These data indeed suggest that amisulpride acts as an agonist at the human cardiac serotonin receptor.

**Supplementary Information:**

The online version contains supplementary material available at 10.1007/s00210-026-05219-7.

## Introduction

**Fig. 1:**
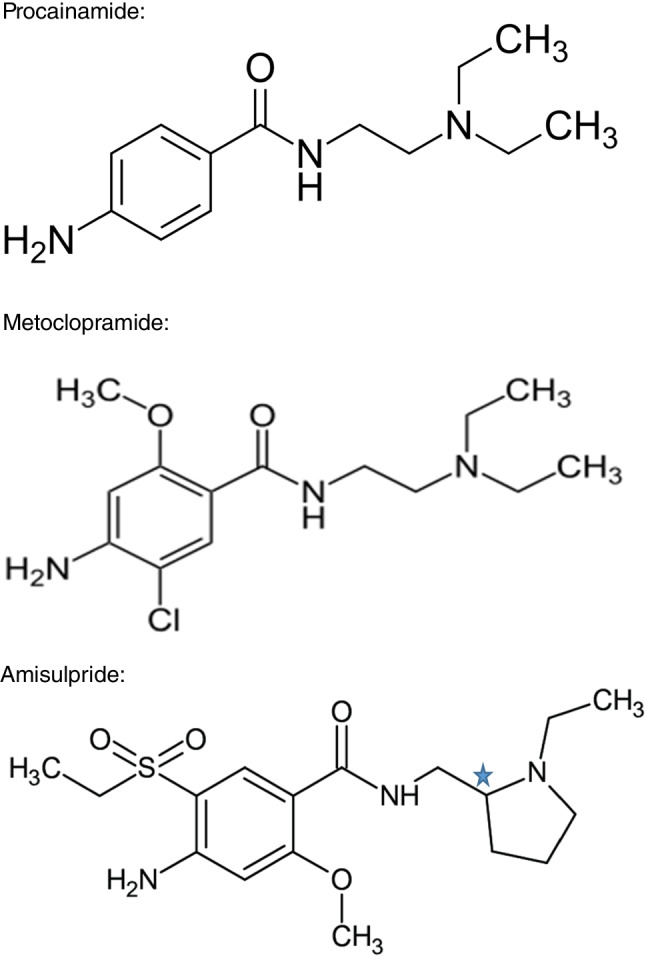
Structure of amisulpride compared to metoclopramide and procainamide. Please note that historically from the starting point procainamide (an antiarrhythmic drug), one went over to metoclopramide (a gastrointestinal drug) and then to the neuroleptic drug amisulpride. The chiral carbon atom in amisulpride is delineated with an asterisk. Please note that, clinically, racemic amisulpride is used

Amisulpride (Fig. 1) is an approved atypical antipsychotic drug (Mortimer 2004, Kang and Shirley 2021, Carli et al. 2021). Amisulpride is used perorally to treat schizophrenia and depression, but recently also (then given intravenously) against post-operative nausea and vomiting (Foong et al. 2020, Hadryś and Rymaszewska 2020, Kang and Shirley 2021, Kishimoto et al. 2023, Zhu et al. 2022). Many benzamides are agonists or antagonists or partial agonists at the human 5-HT_4_ serotonin receptor (Sanger 2009). Amisulpride was initially developed on the basis of the structural formula of the molecule procainamide (an antiarrhythmic drug) and metoclopramide. In contrast to metoclopramide, amisulpride is not primarily given in gastroenterology but in psychiatry (Sneader 2005). Amisulpride was first synthesized in 1975 (Hadryś and Rymaszewska 2020). Thus, amisulpride was developed long before 5-HT_4_ serotonin receptors were functionally characterized or cloned (Dumuis et al. 1989; Gerald et al. 1995). Clinically, and therefore also in the present experimental work, racemic amisulpride is currently being used.

Amisulpride binds to several G-protein-coupled receptors (for instance to D_2_-, D_3_-dopamine receptors or to 5-HT_1_-, 5-HT_2B_-, and 5-HT_7_-serotonin receptors: Abbas et al. 2009, Roth 2025). In other words, we found no evidence in the literature that amisulpride would bind to and thus may act by means of the 5-HT_4_ serotonin receptor in any organ and here our work on the heart started. Our laboratory is specialized in cardiac pharmacology, and this explains the present focus on cardiac side effects of a primarily psychiatric drug, amisulpride.

We had shown before that the direct parent compound of amisulpride, namely, metoclopramide (cf. Figure 1), acted as an agonist at human cardiac 5-HT_4_ serotonin receptors (Neumann et al. 2021a, 2021b). Therefore, we thought amisulpride might behave in a similar way at the human 5-HT_4_ serotonin receptor. These 5-HT_4_ serotonin receptors are of interest in cardiology because all positive inotropic, dromotropic, and chronotropic effects of serotonin in human hearts are 5-HT_4_ serotonin receptor-mediated (e.g., Neumann et al. 2023). Serotonin is present in thrombocytes but also in cardiac muscle and can be formed in the human heart from its precursor (Gergs et al. 2017). The 5-HT_4_ serotonin receptor in the heart leads, besides other effects, to a stimulation of L-type Ca^2+^-channels mediated by activation of the adenylyl cyclases (Ouadid et al. 1992). Such 5-HT_4_ serotonin receptors are functionally lacking in the wild-type mouse heart (WT): Serotonin fails to increase force of contraction and beating rate in isolated hearts or atrial preparations from adult wild-type mice (Gergs et al. 2010, 2013). Therefore, we established a transgenic mouse model with cardiac-specific overexpression of this human 5-HT_4_ serotonin receptor (5-HT_4_-TG). Cardiac preparations of 5-HT_4_-TG but not WT display positive inotropic and positive chronotropic effects to 5-HT_4_ serotonin receptor agonists. 5-HT_4_-TG can therefore be used to study the actions of similar benzamides at the human cardiac 5-HT_4_ serotonin receptor (Gergs et al. 2010; Neumann et al. 2024a, Neumann et al. 2024b; review: Neumann et al. 2017, Neumann et al. 2023).

We asked whether amisulpride would bring about positive inotropic and positive chronotropic effects in these 5-HT_4_-TG and not in littermate wild-type mice (Fig. 1B). One would think that amisulpride might activate the 5-HT_4_ serotonin receptor in the human heart and thereby lead to a rise in force of contraction (Fig. 1B). We found it important also to study the cardiac effect of amisulpride in mice, because intact mice were often used to study effects of amisulpride (e.g., Jahreis et al. 2023, Schreiber and Pick 2021, Donahue et al. 2017). Therefore, one would like to know what cardiac effects amisulpride might have elicited in these mouse models. Based on these findings in mice, we finally tested the contractility in human atrial preparations (HAP) to achieve our ultimate goal: to understand cardiac (side) effects of amisulpride in man.

In summary, we mainly tested the following claims:

1. Amisulpride raised force of contraction in atrial preparations from 5-HT_4_-TG but not wild-type mice.
2. Amisulpride heightened force of contraction in human atrial preparations via 5-HT_4_ serotonin receptor.

## Materials and methods

### Transgenic mice

Transgenic mice were generated several years ago (Gergs et al. 2010). The handling and breeding of the animals complied with all pertinent regulations (permission # I8M9). At the day of the contraction experiment, the animals were adults that means here about 170 days of age. Both sexes were studied. We did not note any sex differences in the drug responses and therefore the data were pooled.

### Contractility studies in mice

Animals were sacrificed by cervical dislocation as described before (Gergs et al. 2010). The atrial preparations were pierced by metal hooks at each end. The bathing solution, a modified Tyrode’s solution (buffer), present in the organ baths (10 ml volume) contained 119.8 mM NaCl, 5.4 mM KCl, 1.8 mM CaCl_2_, 1.05 mM MgCl_2_, 0.42 mM NaH_2_PO_4_, 22.6 mM NaHCO_3_, 0.05 mM Na_2_EDTA, 0.28 mM ascorbic acid, and 5.05 mM glucose. Ascorbic acid is routinely present in our solution to impair oxidation of drugs, for example, isoprenaline. The solution was continuously gassed with 95% O_2_ and 5% CO_2_ and maintained at 37 °C and pH 7.4 (Neumann et al. 1998). We always stimulate left atria electrically in this setup. The stimulator (Grass SD 9, Quincy, Massachusetts, USA) generated a rectangular biphasic direct current with 5-ms duration and a height of 3 to 5 V just sufficient to initiate muscle contraction. Muscles were stretched such that the force generation was maximum. The signals from the force transducer were relayed to a bridge amplifier (ADInstruments, Oxford, England). Then, the signals were digitized (PowerLab, ADInstruments) and fed into a personal computer (Dell, Halle, Germany). The signals were analyzed with the help of commercial software (PowerLab 8 from ADInstruments): We had configured the software such that we could read out the force of contraction in milliNewton (mN) and the maximum and minimum first derivatives to time of the force (dF/dt). This provides the maximum rate of tension development and the maximum rate of relaxation (mN/s), respectively. The rate of tension development can behave different from the force. For instance, in hyperthermia, muscles contract more slowly, and an increase in force can occur without an increase in the rate of tension development (e.g., Hiis et al. 2018). Positive inotropic drugs usually induce more potently a change in the rate of force development than in the generated force. This is explained with the assumption that the intrinsic contractility of a muscle is more closely related to the rate of tension development (e.g., Braunwald et al. 1967). After equilibration was reached, amisulpride was added to the contracting muscles in the organ baths.

### Contractile studies on human preparations

The contractile studies on human atrial preparations were done using the same setup and buffer as used in the mouse studies (see preceding section). We stretched the muscle to maximum display of force. Stimulation rate was identical (1 Hz). Stimulation voltage was usually around 5 to 10 V, approximately 10% higher than the voltage at which atrial contraction was initiated. Atrial strips were cut also with the help of a dissecting microscope. The atrial strips were cut from the interior side of the right atrial strips we took from the surgical theater. The samples were obtained from the right atrium of the patient near the cannulation site for extracorporeal circulation. The atrial strips were transported within 30 min from the surgical theater to the institute in plastic flasks containing the bathing buffer (as in the mouse experiments) at room temperature. The samples were obtained from seven male patients, aged 57–81 years. Cardiac drug therapy included metoprolol or a similar β-adrenoceptor antagonist, furosemide or a similar loop diuretic drugs, apixaban or a similar anticoagulant and acetylsalicylic acid. Cardiac comorbidities included hypertension and heart failure. Other morbidities were diabetes type 2, obesity, lung disease, and prostatomegaly. Our methods used for atrial contraction studies in human samples have been previously published and were not altered in this study (Gergs et al. 2009, 2017, 2023).

### Data analysis

Data in the graphs of this project show means ± standard error of the mean. Statistical significance was estimated using the analysis of variance followed by Bonferroni’s *t*-test or the paired Student *t*-test as appropriate as delineated in detail in the appropriate figure legend. We defined a probability value smaller than 0.05 as significant in the present study. For the purpose of testing for normality, statistical analyses, and graphical presentation of data, the software Prism9 (GraphPad Software, San Diego, California, USA) was used.

### Drugs and materials

The drugs isoprenaline-hydrochloride, racemic amisulpride, or cilostamide (N-cyclohexyl-N-methyl-4-(1,2-dihydro-2-oxo-6-quinolyloxy)butyramide) were obtained from Merck, Dreieich, Germany. GR125487 (5-fluoro-2-methoxy-[1-[2-[(methylsulfonyl)amino]ethyl]−4-piperidinyl]−1H-indole-3-methylcarboxylate sulfamate and cilostamide (6-[3-(N-cyclohexyl-N-methylcarbamoyl)propoxy]quinolin-2[1H]-one) were from Tocris via Bio-Techne (Wiesbaden, Germany). All other chemicals were of the highest purity grade commercially available. Deionized water was used throughout the experiments for preparation of modified Tyrode’s solution. Stock solutions were prepared fresh daily.

## Results

**Fig. 2:**
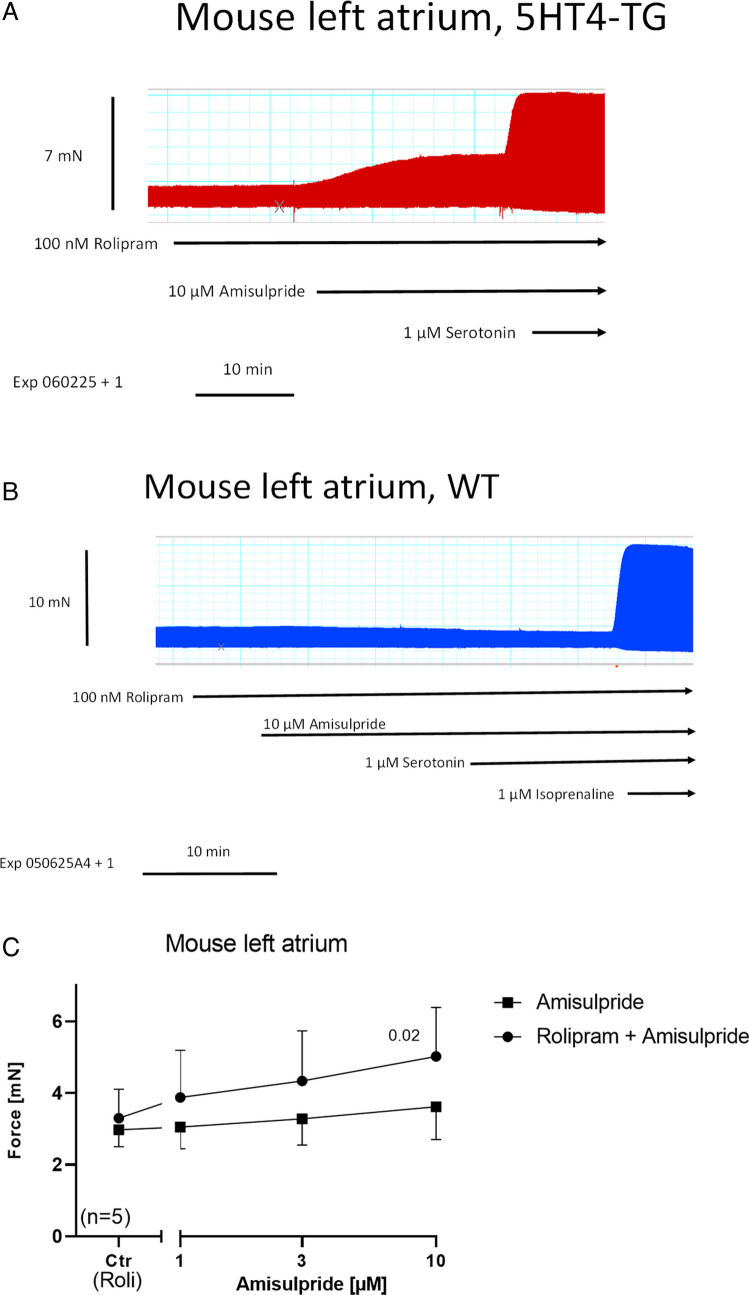
Amisulpride increased force in 5-HT_4_-TG. Original recordings of force in mouse left atrial preparation from 5-HT_4_-TG. In the presence of rolipram, amisulpride (**A**) induced a time-dependent positive inotropic effect in 5-HT_4_-TG. Subsequently, serotonin (1 µM) was given. **B** Original recordings in WT; subsequently, isoprenaline (1 µM) was applied. Concentration–response curve for the effect of amisulpride, cumulatively applied on force of contraction in mouse left atrial preparation from 5-HT_4_-TG in the absence (**C**, squares) and in the presence (**C**, circles) of 100 nM rolipram (Roli). *p* values are listed above the data points versus Ctr using a paired *t*-test. Abscissae: negative decadic logarithms of molar drug concentrations (**C**). Number in brackets indicates number of experiments. Horizontal bars indicate time axis in minutes (min)

To detect a clear effect of amisulpride, we pre-treated the atrial preparations from the mouse with the phosphodiesterase IV inhibitor rolipram at 100 nM as in previous reports (Neumann et al. 2019, 2024a, 2024b; Hesse et al. 2024; Neumann et al. 2025a, b). This led to the following result, as depicted in an original tracing of the left atrial preparations from 5-HT_4_-TG (Fig. 2A). In the experiment, we first gave 100 nM rolipram and additionally applied 10 µM amisulpride, which increased the force of contraction. Thereafter, we added 1 µM serotonin, which increased the force of contraction further. This concentration of serotonin, as we know from previous studies, maximally increased the force of contraction in left atria from 5-HT_4_-TG (Gergs et al. 2013). In the left atrium of WT, amisulpride and serotonin failed to increase the force of contraction in the presence of rolipram (Fig. 2B). The force of contraction after 10 µM amisulpride was 97% ± 7% of that before the addition (*p* = 0.53, *n* = 5). However, the left atrial preparation from WT displayed a positive inotropic response to 1 µM isoprenaline that was studied here as a positive control. We also established concentration–response curves to amisulpride (1, 3, and 10 µM) in the left atrial preparations from 5-HT_4_-TG. In the absence of rolipram, there was only a tendency to higher mean values of force, which never gained statistical significance (Fig. 2C). However, in the presence of 100 nM rolipram, amisulpride caused a concentration-dependent increase in the force of contraction. Amisulpride significantly increased the force of contraction at 10 µM (Fig. 2C). Although the data are not shown, the increase in the force of contraction of 10 µM amisulpride is significantly lower than that of 1 µM serotonin under the present experimental conditions (data are not indicated, cf. Figure 2A). In further experiments, we focused on 10 µM amisulpride.

**Fig. 3:**
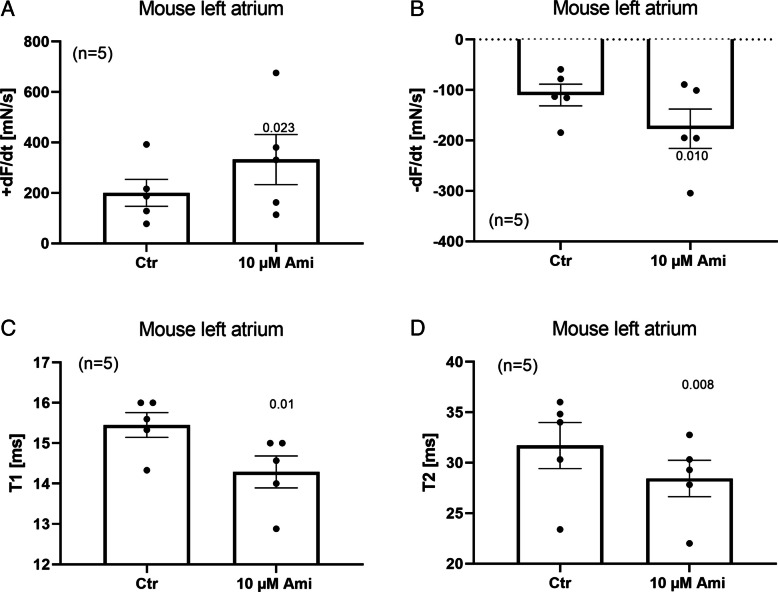
Amisulpride altered contractile parameters in 5-HT_4_-TG. Amisulpride increased the rate of tension development (**A**) and the rate of tension relaxation (**B**). Amisulpride reduced the time to peak tension (**C**) and the time of relaxation (**D**). Ordinates indicate force in milliNewton per second (mN/s) (**A** and **B**) or time in milliseconds (ms, **C** and **D**). *p* values are listed above the data points versus Ctr using a paired *t*-test. Number in brackets indicates number of experiments

In the presence of rolipram, 10 µM amisulpride increased the rate of tension development (Fig. 3A) and augmented the rate of tension relaxation (Fig. 3B). In the presence of rolipram, 10 µM amisulpride reduced the time to peak tension (T1: Fig. 3C) and the time of relaxation (T2: Fig. 3D).

**Fig. 4:**
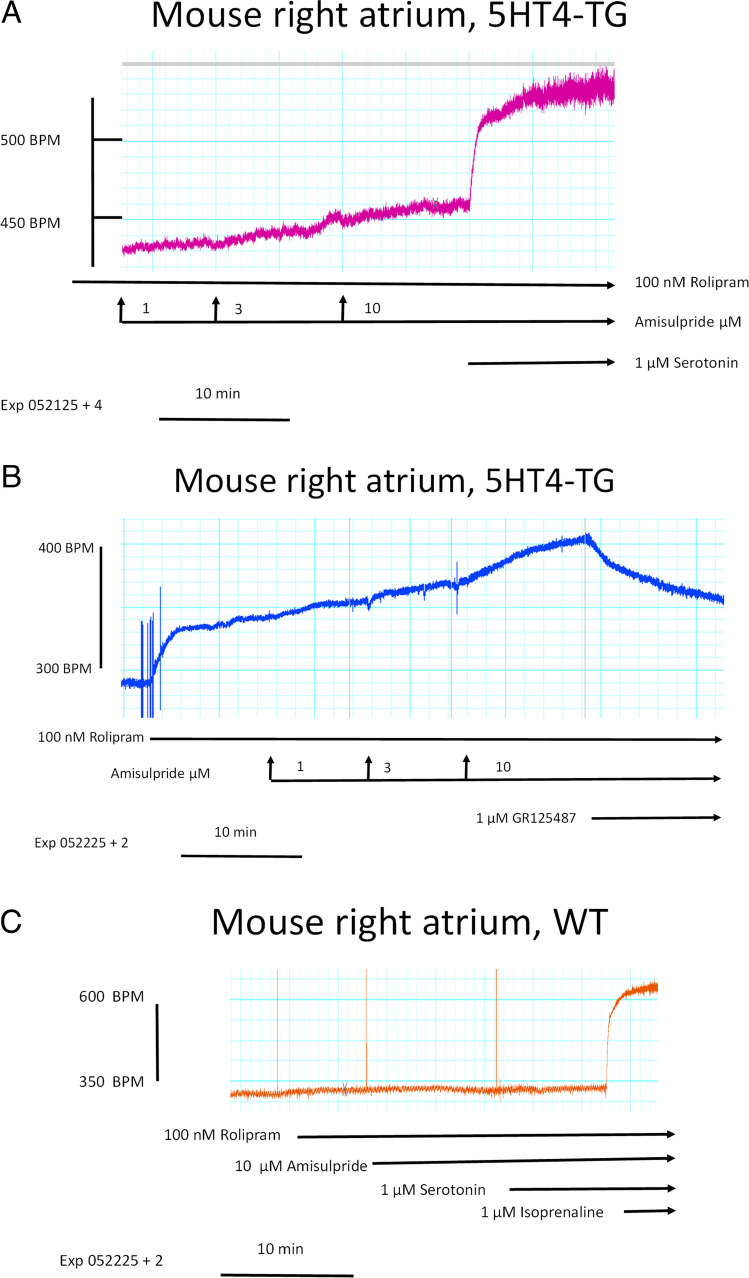
Amisulpride increased heartbeat in 5-HT_4_-TG. In right atrial preparations from 5-HT_4_-TG, in the presence of 100 nM rolipram, amisulpride concentration- and time-dependently increased the beating rate. This is seen in an original recording (**A**). Subsequently applied serotonin increased the beating rate further (**A**). This positive chronotropic effect of amisulpride is antagonized by the 5-HT_4_ serotonin receptor antagonist GR125487 (**B**). In contrast, in a right atrial preparation from WT, amisulpride as well as serotonin failed to increase the beating rate. But isoprenaline, used here as a positive control, increased the beating rate: original recording in (**C**). Horizontal bars indicate time axis in minutes (min). Vertical lines indicate the beating rate in beats per minute (BPM)

Interestingly, amisulpride, in the presence of 100 nM rolipram, concentration- and time-dependently increased the beating rate in the isolated spontaneously beating right atrial preparations from 5-HT_4_-TG (original recording: Fig. 4A). 10 µM amisulpride increased the beating rate to 123% ± 15% compared to the basal beating rate in the presence of rolipram (*p* = 0.023, *n* = 5). This effect was reversed by the 5-HT_4_ serotonin receptor antagonist GR125487 (Fig. 4B). Data from several experiments showed that GR125487 (1 µM) reduced the beating rate to 56% ± 8% of the value reached by 10 µM amisulpride (*p* = 0.014 vs. Ctr, *n* = 5). Of note, 1 µM GR125487 has no effects on its own in this model (Gergs et al. 2010). In contrast, in right atrial preparations from WT, in the presence of 100 nM rolipram, 10 µM amisulpride and also 1 µM serotonin failed to raise the beating rate. However, 1 µM isoprenaline, again used as a positive control, augmented the beating rate (original recording: Fig. 4C). To summarize, the results of several experiments indicate that 10 µM amisulpride, over a period of 10 min, resulted in a heart rate that was 104% ± 11% of that observed after rolipram (*p* = 0.043, *n* = 5). Any effects of amisulpride in right atrial preparations from WT were not further studied.

**Fig. 5:**
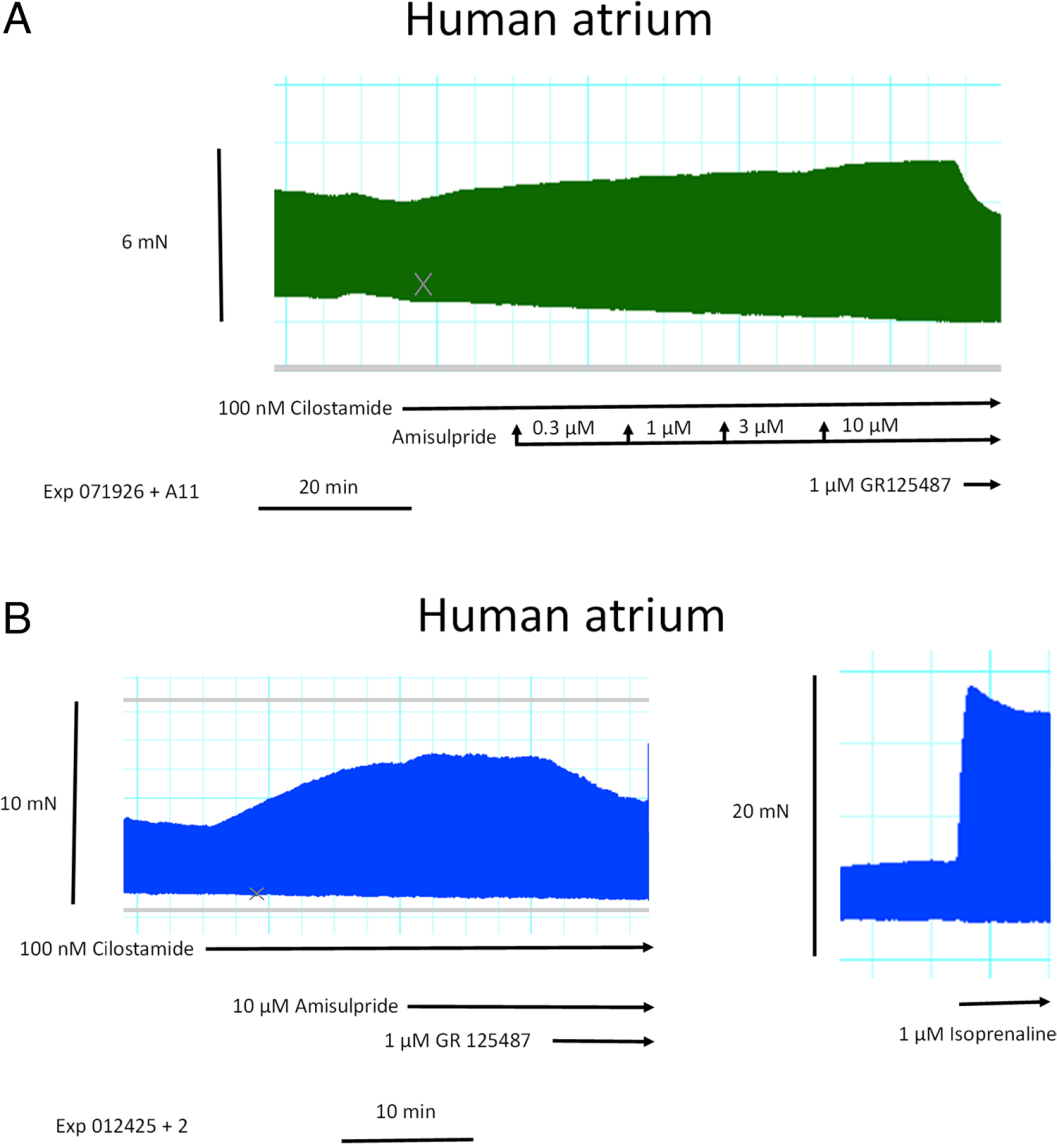

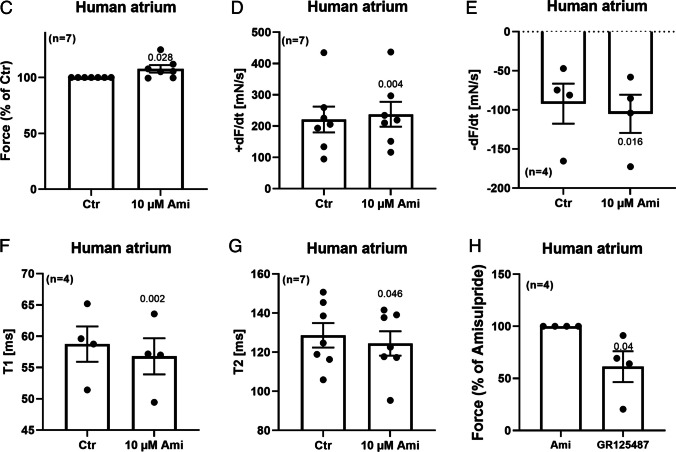
Amisulpride increased force in the human atrium. Original recording of the concentration–response and time-dependent positive inotropic effect of amisulpride (**A**) in the presence of 100 nM cilostamide in milliNewton (mN) in electrically stimulated human right atrial muscle strips. Original recording of the positive inotropic effect of 10 µM amisulpride (**B**) in the presence of 100 nM cilostamide in milliNewton (mN) in electrically stimulated human right atrial muscle strips. Additionally, applied 1 µM GR125487 reduced force of contraction (**A** and **B**). Additionally applied 1 µM isoprenaline increased force of contraction (**B**). Summarized data are depicted in (**C)** for force of contraction in percentage of pre-drug value. In (**D**), the maximum rate of contraction (+ dF/dt) and, in (**E**), the maximum rate of relaxation (− dF/dt) are given. In (**F**), the time to peak tension (T1) and, in (**G**), the time of relaxation (T2) are depicted. In (**H**), the negative inotropic effect of 1 µM GR125487 on force of contraction in the presence of 100 nM cilostamide and 10 µM amisulpride (Ctr) in HAP is plotted. In detail, we waited until the positive inotropic effect of 10 µM amisulpride in the presence of 100 nM cilostamide had reached a plateau and then the 5-HT_4_ serotonin receptor antagonist GR125487 was added. Ctr values in (**H**) amounted to 8.70 ± 1.11 (*n* = 4). *p* values are listed above the data points versus Ctr using a paired *t*-test. Numbers in brackets mean number of experiments. Horizontal bars indicate time axis in minutes (min)

Next, we wanted to test the effects of amisulpride in the human heart, more specifically in the isolated human atrium. To this end, we studied isolated electrically stimulated human atrial preparations (HAP) in the organ bath. We studied the effects in the presence of 100 nM cilostamide (phosphodiesterase 3 inhibitor) to accentuate effects of 5-HT_4_ serotonin receptor agonists. Under the presence of cilostamide, amisulpride (0.3 to 10 µM) concentration-dependently augmented the force of contraction in HAP (original tracing: Fig. 5A). However, the concentration-dependent response was not strong and we decided therefore to focus on 10 µM amisulpride in HAP. A typical recording is seen in Fig. 5B (left hand side). After wash-out, we added 1 µM isoprenaline, which was much more effective (Fig. 5B) than amisulpride in the presence of cilostamide. Please note that in Fig. 5B, all tracings are from the same HAP and therefore comparable. Such actions are summarized in Fig. 5C. In the presence of 100 nM cilostamide, 10 µM amisulpride significantly increased force of contraction (Fig. 5C) and the rate of tension development (Fig. 5D) and augmented the rate of tension relaxation (Fig. 5E). In addition, under these conditions, 10 µM amisulpride reduced the time to peak tension (T1: Fig. 5F) and the time of relaxation (T2: Fig. 5G). Furthermore, we show in the original recordings that the positive inotropic effects of amisulpride in HAP were clearly reversed by 1 µM GR125487 (Fig. 5A,B, left panel). Several such experiments supported that the positive inotropic effects of amisulpride were significantly reversed by GR125487 (*p* = 0.04, *n* = 4, Fig. 5H). We have shown before that 1 µM GR125487 has no effects on its own in HAP (Neumann et al. 2025b). These findings indicate that the positive inotropic effects of amisulpride are 5-HT_4_ serotonin receptor-mediated in HAP and that amisulpride is an agonist at the human cardiac 5-HT_4_ serotonin receptor. After wash-out of GR125487 or addition of 1 µM isoprenaline, the contraction of the HAP is recovered or increased again, which certificated a proper physiological response of HAP (Fig. 5B, right panel).

## Discussion

### Main new findings

The main new finding was amisulpride, an antipsychotic agent, can exert chronotropic and inotropic actions in the atrium as a functional agonist of the human 5-HT_4_ serotonin receptor. This finding is plausible when one takes into consideration that amisulpride is structurally a benzamide (Fig. 1). Other benzamides such as metoclopramide (Fig. 1) are 5-HT_4_ serotonin receptor agonists (Villalon et al. 1991). In addition, like amisulpride, metoclopramide is also a D_2_-dopamine receptor antagonist (Martres et al. 1984). In contrast to amisulpride, one apparently never found that metoclopramide is an antipsychotic drug (Stanley et al. 1980). At least in the left atrium of 5-HT_4_-TG, 30 µM procainamide (Fig. 1) also had a positive inotropic effect in the presence of 100 nM rolipram (supplementary data 1), which is consistent with the view that benzamides in general are agonistic at human 5-HT_4_ serotonin receptors.

### Mechanism of amisulpride

Amisulpride acts as an agonist at the human cardiac 5-HT_4_ serotonin receptor in 5-HT_4_-TG because amisulpride increased contractility in the left atrium from 5-HT_4_-TG and not in the left atrium from WT. We had overexpressed the human 5-HT_4_ serotonin receptor in 5-HT_4_-TG. Thus, it is likely that amisulpride is an agonist at the human cardiac serotonin receptor in these transgenic mice. Amisulpride increased the beating rate in the right atrium of 5-HT_4_-TG and not of WT. This suggests that the human 5-HT_4_-serotonin receptor in the sinus node of the 5-HT_4_-TG is activated by amisulpride. This assumption is supported by the fact that a 5-HT_4_ serotonin receptor antagonist, 1 µM GR125487, reversed the positive chronotropic effect of amisulpride. We have shown previously that GR125487 reversed the positive chronotropic effect of serotonin in isolated perfused hearts but also in isolated right atrial preparations from 5-HT_4_-TG but was inactive in WT (Gergs et al. 2010, 2013). There is agreement in the literature that stimulation of the 5-HT_4_ serotonin receptor leads to an increase in the phosphorylation state of proteins that are substrates for the cAMP-dependent protein kinase in all tissues where the receptor is expressed. We and others described that serotonin via the 5-HT_4_ serotonin receptor can increase the phosphorylation state of phospholamban in HAP, of the inhibitory subunit of troponin, elevates the Ca^2+^ transients in cardiomyocytes, and increases the current through L-type calcium channels in the mammalian heart (human atrium: Gergs et al. 2009, Christ et al. 2014; 5-HT_4_-TG: Gergs et al. 2010). Therefore, the increase in the force of contraction and the faster relaxation rate and shorter relaxation time that we observed in mice (Fig. 3) with amisulpride can be explained, at least in part, by phosphorylation of phospholamban. But also other targets like the troponin I or the L-type calcium channels should play a role because they are targets of the cAMP-dependent protein kinase and are activated by phosphorylation.

Carefully comparing the effects of amisulpride on developed force or its first derivatives in transgenic (Figs. 2 and 3) or human atria (Fig. 5), one may notice that the effects in transgenic mice are more pronounced than in human atria. We explain this apparent discrepancy by the assumption that the expression of the transgenic human 5-HT_4_ serotonin receptor is higher in the atrium in the transgenic mice than the native 5-HT_4_ serotonin receptor in the human atrium. We have noticed this before with other 5-HT_4_ serotonin receptor agonists (e.g., Neumann et al. 2021a; Hesse et al. 2024).

Amisulpride acted as an agonist at the 5-HT_4_ serotonin receptor in the isolated human atrium in the presence of cilostamide. These findings are in agreement with an action of amisulpride via an increase in cAMP production in the human atrium as cilostamide is thought to inhibit the degradation of cAMP that had been formed via stimulation of the 5-HT_4_ serotonin receptor. The effects of amisulpride seem to be mediated via the human 5-HT_4_ serotonin receptor because they are reversed by GR125487 (Fig. 5H), an antagonist at the 5-HT_4_ serotonin receptor in HAP. From this, we conclude that amisulpride may exert positive inotropic and positive chronotropic effects in the human heart, which could be mediated by the human 5-HT_4_ serotonin receptor.

### Limitations of the study

Clearly, it might be possible to understand the molecular correlations of our findings better by performing radioligand binding studies with amisulpride at recombinant human 5-HT_4_ serotonin receptors or even in human cardiac membranes. We failed to perform ligand binding studies in hearts from 5-HT_4_-TG (Gergs et al. 2010). Others found very small signals for ligand binding at the human 5-HT_4_ serotonin receptors in the human atrium (much less than β-adrenoceptor binding: Kaumann et al. 1996). Theoretically, one could nowadays assess the crystal structure of the human 5-HT_4_ serotonin receptor. But that is a task for future studies for specialized laboratories. One can argue that we have not tested the effects on the sinus node of man directly but as a surrogate only used the mouse right atrium. However, in humans, the injection of serotonin increased the beating rate (Le Messurier et al. 1959). Serotonin augmented the current through the pacemaker channels in isolated human atrial muscle preparations (Pino et al. 1998). Thus, it is reasonable to assume that the same receptor acts in mice and men to mediate an increase in the heart beat after serotonin application and this pathway may be used by amisulpride in man. The effects of amisulpride as agonist only became apparent when a phosphodiesterase inhibitor, cilostamide, was given. In the absence of cilostamide, we failed to measure a positive inotropic effect (data not shown).

In summary, in the presence of phosphodiesterase inhibitors, amisulpride enhanced both the force of contraction and beating rate in the atrial preparations from 5-HT_4_-TG mice and increased the force of contraction in the human atrial preparations via the human 5-HT_4_ serotonin receptor.

## Supplementary Information

Below is the link to the electronic supplementary material.

ESM1 (DOCX 455 KB)

## Data Availability

The data of this study are available from the corresponding author upon reasonable request.
