# Supplementary material for: Positive inotropic and chronotropic effects of amisulpride via stimulation of 5-HT4 serotonin receptors in the isolated atrium from mouse and humans
Source: Naunyn Schmiedebergs Arch Pharmacol. 2026 Mar 21;399(9):13197–207. doi: 10.1007/s00210-026-05219-7 (PMC13357458; doi:10.1007/s00210-026-05219-7)
Supplement: Supplementary file 1 — (DOCX 455 KB) [file 210_2026_5219_MOESM1_ESM.docx]

Supplementary data 1:

Procainamide increases force of contraction

Original recordings of force in mouse left atrial preparation from 5-HT_4_-TG. In the presence of rolipram, procainamide concentration and time-dependently induced a time-dependent positive inotropic effect in 5-HT_4_-TG. This effect was reversed by GR125487. The effect of rolipram was abolished by subsequent wash out, indicating that the drug effects were reversible. Horizonal bar indicates time axis in minutes (min). Ordinate indicates force in milli Newton.

Legend ot supplementary data 1: First 100 nM
